# geoBoundaries: A global database of political administrative boundaries

**DOI:** 10.1371/journal.pone.0231866

**Published:** 2020-04-24

**Authors:** Daniel Runfola, Austin Anderson, Heather Baier, Matt Crittenden, Elizabeth Dowker, Sydney Fuhrig, Seth Goodman, Grace Grimsley, Rachel Layko, Graham Melville, Maddy Mulder, Rachel Oberman, Joshua Panganiban, Andrew Peck, Leigh Seitz, Sylvia Shea, Hannah Slevin, Rebecca Youngerman, Lauren Hobbs

**Affiliations:** 1 Department of Applied Science, William & Mary, Williamsburg, Virginia, United States of America; 2 Geospatial Evaluation and Observation Lab, William & Mary, Williamsburg, Virginia, United States of America; 3 Intel Corporation, Santa Clara, California, United States of America; 4 Booz Allen Hamilton, McLean, Virginia, United States of America; 5 Harvard School of Public Health, Cambridge, Massachusetts, United States of America; 6 Deloitte, Arlington, Virginia, United States of America; University of North Carolina at Charlotte, UNITED STATES

## Abstract

We present the geoBoundaries Global Administrative Database (geoBoundaries): an online, open license resource of the geographic boundaries of political administrative divisions (i.e., state, county). Contrasted to other resources geoBoundaries (1) provides detailed information on the legal open license for every boundary in the repository, and (2) focuses on provisioning highly precise boundary data to support accurate, replicable scientific inquiry. Further, all data is released in a structured form, allowing for the integration of geoBoundaries with large-scale computational workflows. Our database has records for every country around the world, with up to 5 levels of administrative hierarchy. The database is accessible at http://www.geoboundaries.org, and a static version is archived on the Harvard Dataverse.

## Introduction

**Fig 1:**
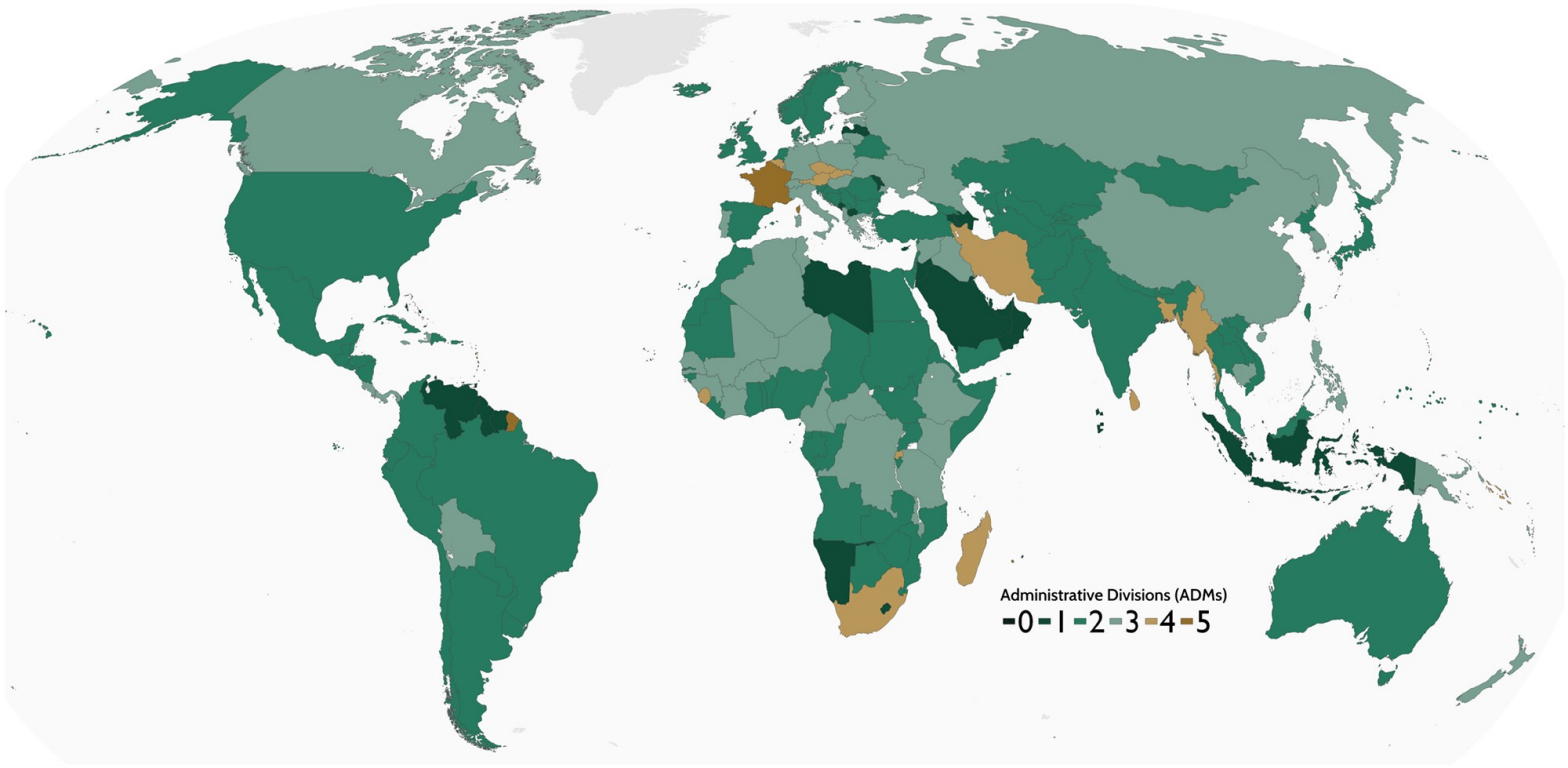
Current state of the geoBoundaries database. All countries are shaded to indicate the depth of hierarchy of the administrative zones collected. Higher numbers indicate deeper hierarchies are available.

The geoBoundaries Global Administrative Database (geoBoundaries) is an online, open license data resource which contains the geographic boundaries of administrative divisions (i.e., states and counties) for every country in the world (see Fig 1). The database is standardized using ISO 3166-1 alpha-3 encoding, and every boundary has a globally unique ID, allowing for integration with large-scale computational workflows. The database is not intended for visualization, but rather for scientific inquiry in which the highest level of precision available is desired. Further, we integrate boundaries exclusively with licenses highly permissive for scientific inquiry, and provision a full data lineage for each of our underlying files.

Studies leveraging subnational units of observations—such as districts, census blocks, counties, or other subdivisions—are common across the health, computational and social sciences (for a few recent examples, see [1], [2], and [3]). Paradoxically, interest in subnational research has not been accompanied by intensive collection efforts focused around subnational administrative boundaries. Only a small collection of groups (see, for example, [4–6, 7]) have sought to collect or provision administrative boundaries; however, to date no organization has focused on the provision of highly precise, open license data for scientific use and research replication. This is the result of a range of factors, most predominant of which is the lack of clear license terms attributable to most boundary datasets currently available in open environments.

We view open, highly precise information on geographic boundaries as critical for research both within academia and the broader scientific community. The lack of open boundary information around the world results in researchers being unable to answer critical questions that would otherwise be highly valuable—i.e., answering “What is the accessibility of clinics in the Luapula province in Zambia?” requires not only a source of information such as road networks, but also a precise shape defining the boundary of the Luapula province. The geoBoundaries dataset preferences the most precise information available at the cost of usability, contrasted to alternative boundary data products that seek to promote usability at the cost of precision (see, for example, [7]). This decision results in exceptionally large files relative to alternative databases, but can also provide higher accuracy for applications that demand it.

As is detailed below, we further focus on provisioning the highest quality dataset feasible for each individual country; this results in a preference for within-country validity of topology, with no guarantee of cross-country topology validity. In practice, this ensures that boundaries share the same lines within each country, but it is possible for national boundaries to overlap one another. For example, in cases where two nations share a contested border, we might rely on each countries definition of their own boundaries—thus potentially resulting in an overlapping case.

To the authors’ knowledge, the geoBoundaries database is also the only global administrative database that is provisioned with a full quality assurance procedure, including manual revisions and hand digitization of physical maps where appropriate. Nightly build scripts are performed which provide for a wide range of automated quality checks—including if the source website(s) can be accessed, topology validity, file validity, and more. In cases where any element of the build fails, geoBoundaries practitioners work in a collaborative, multi-stakeholder environment to identify, fix or replace boundaries that require attention. Subversions are used to indicate changes; a full lineage of all geoBoundaries versions is retained in online repositories.

We note the database presented here can mitigate challenges associated with the replication of future studies. Because of the closed- or unknown-license nature of other administrative zone databases, researchers are frequently precluded from legally distributing underlying boundary information with any replication data packages. By provisioning an open data source with full license detail for every boundary, geoBoundaries allows any researcher to confidently redistribute all boundaries used in an analysis. The rest of this piece details our methodology for collection, correction, and provision of administrative boundaries.

## Materials and methods

We have collected the latitude and longitude coordinates used to define the boundaries of political administrative boundaries for every country in the world, and provision these in both a static [8] and regularly updated [9] form. Building on numerous efforts within the geographic community to establish frameworks for the collection and dissemination of geographic data [10], we adopt a multi-stage procedure to construct this information. While we will go into further detail for each stage, they can be broadly defined as:

1. Data collation
  - Identify the legal authority or authorities that define the latitude and longitude demarcations of administrative boundaries within a country.
  - Contact this authority (digitally, over phone or in person) to ascertain the location or existing definitions of boundaries, and if they exist in digital form or not.
  - If no open licensed representation (physical or digital) is available from the authority or authorities responsible for boundary definition, conduct a search across alternative data providers (inclusive of physical maps) to identify open licensed alternatives.
  - Collect all required metadata, inclusive of data lineage, license, year, and other elements summarized in Table 1.
  - If necessary, hand-digitize physically mapped documents.
2. Topology & Related Data Quality & Cleaning Techniques
  - Manual correction of missing entities and multi-source integration.
  - Semi-manual standardization of projections to WGS-84.
  - Manual & Automated identification and correction of internal topological errors.
  - Automated identification of errors in recorded metadata, including a wide range of license and other validations.
  - Automated identification of errors in file structure.
3. Data provision
  - Automated build scripts create a unified, hierarchical structure for all administrative zones within each country.
  - A variety of common spatial data file formats are created for each countries administrative boundaries.
  - Automated metadata is produced for each data product.
  - All data is made available through both a static, machine-parseable interface and API at www.geoboundaries.org.

**Table 1:** Minimal data schema for geoBoundaries files. All fields noted in this table must be collected and validated for inclusion in a release. *URLs provided as exemplars only; within the database, full paths to exact landing pages from which data was retrieved are included.

Field Name	Description	Type	Example	Unique Vals.
boundaryID	A unique ID created for every boundary in the geoBoundaries database by concatenating ISO 3166-1 3 letter country code, boundary level, geoBoundaries version, and an incremental ID.	String	‘AFG-ADM1-2-0-0-G1’	632
boundaryISO	The ISO 3166-1 3-letter country codes for each boundary.	String	‘ARM’	198
boundaryYear	The year for which a boundary is representative.	Integer	2018	18
boundaryType	The type of boundary defined.	String	‘ADM 1’	10
boundarySource-K	The name of the *K*^*th*^ source for the boundary definition used (with most boundaries having two identified sources).	String	‘Government of Armenia’	189
boundaryLicense	The specific license the data is released under.	String	‘Creative Commons Attribution 4.0 International (CC BY 4.0)’	24
licenseDetail	Any details necessary for the interpretation or use of the license noted.	String	‘Free to adapt and redistribute’	58
licenseSource*	A resolvable URL (checked at the time of data release) declaring the license under which a data product is made available.	String	‘http://www.stats.govt.nz’	145
boundarySourceURL*	A resolvable URL (checked at the time of data release) from which source data was retrieved.	String	‘http://geo.stp.gov.py’	136
boundaryUpdate	A date encoded following ISO 8601 (Year-Month-Date) describing the last date this boundary was updated, for use in programmatic updating based on new releases.	String	‘2019-12-23’	23
downloadURL	A URL from which the geoBoundary can be downloaded.	String	See Fig 2	632

### Data collation

We follow a multi-stage procedure for the identification, assessment, and selection of products to include within the geoBoundaries database. All boundaries are validated by at least two practitioners in this process.

The first stage of the collation process is to identify the legal authority (or authorities) that define latitude and longitude demarcations of administrative boundaries within a country. Because we preference within-country sources, we then contact this authority to acquire relevant data for inclusion into the database. If the authority identified does not have or is unable to provide an open licensed representation of boundaries within their country, we proceed to search across alternative data providers—including archival library searches for physical maps. In the case of multiple, competing alternative data providers, we select mapped representations which are supported by multiple alternative sources. In rare cases where no digital representation is available, we hand-digitize mapped documents for inclusion, relying on the physical document in question for relevant license and metadata.

The second stage of collation involves identifying all relevant metadata, inclusive of data lineage, license(s), and other items seen in Table 1. In many cases, this may involve contacting individuals or groups for appropriate license information; in these cases, personal communications providing permission for use are archived on a publicly available website.

### Topology & related data quality & cleaning techniques

For each public version of geoBoundaries, a rigorous set of semi-automated quality checks and corrections are conducted. First and foremost, all metadata associated with each boundary is confirmed to be accurate and valid by at least two practitioners and an automated script. This includes ensuring each file name adheres to the schema noted in Table 1; all files have valid ISO-3166-1 Alpha 3 codes; all boundaries have a source and open license (currently accepted licenses are described in Table 3). Further, at the time of build we ensure that all URLs in the database are resolvable, including source and license.

In addition to metaData, a number of topological corrections are performed on each boundary to ensure within-country topological consistency. This is conducted in a two stage process. Stage 1 is a manual stage in which the shape boundary itself is examined for any large-scale inconsistency (i.e., gaps or holes between regions due to missing information); any identified inconsistencies are manually corrected. The second stage of the process is an automated topology operation designed to fix small issues due to errors in measurement precision—for example, if the banks of rivers “cross”. This procedure is implemented using the GEOS software package, identifying and saving the latitude and longitude coordinates (nodes) necessary to recreate a given shape given a certain level of precision (this is implemented as a “zero buffer” operation; while not guaranteed to fix all topology errors, it provides for an algorithmic approach to correcting many common inconsistencies [11]). After these corrections, a check for valid topology is conducted for each set of boundaries, where the definition of validity follows the Open Geospatial Consortium Implementation Standards [12]. Finally, all sets of boundaries are converted to MultiPolygon types for intra-database consistency.

### Data provision

Recognizing that ease of access to high quality datasets is frequently a barrier to use, and that different users may have different technical standards and needs, we have adopted a dynamic workflow which produces a range of both machine and human-readable data formats. Further, within this step we ensure that every boundary within our database has unique identifiers, is available in a structured format, and full data providence for any single shape can always be traced.

The first stage of our data provision pipeline is to enter each boundary into a unified, hierarchical structure. To do this, first every unique Boundary Group and Boundary Type combination (see Table 1) is identified. For each of these boundary groups, an on-disk storage folder is created, and the destination for that location is saved in memory (we will refer to this path as *P*_*i*_, where every boundary group is represented by an index *i*).

Next, we create a unified schema for all individual files, ensuring that the metadata provided for any individual shape is the same across all shapes. This schema is described in full in Table 2. This includes the construction of an ID that will always be unique across all shapes in this and future releases.

**Table 2:** Data schema for individual shapes in geoBoundaries. Fields denoted with a * must be populated for inclusion into the database; other fields are considered optional. Some fields are replicated from the data schema for geoBoundaries files, so that users do not need to join different files for common use cases.

Field Name	Description	Type	Example
shapeID*	The boundary ID, followed by the letter ‘B’ and a unique integer for each shape which is a member of that boundary.	String	‘AFG-ADM1-2-0-0-B1’
shapeName	The identified name for a given shape. ‘None’ if not identified.	String	‘Atome’
shapeGroup*	The country or similar organizational group that a shape belongs to, in ISO 3166-1 where relevant.	String	‘AFG’
shapeType*	The type of boundary represented by the shape.	String	‘ADM 1’
shapeISO	ISO codes for individual administrative districts, where available. Where possible, these conform to ISO 3166-2, but this is not guaranteed in all cases. ‘None’ if not identified.	String	‘AF-SAM’

**Fig 2:**
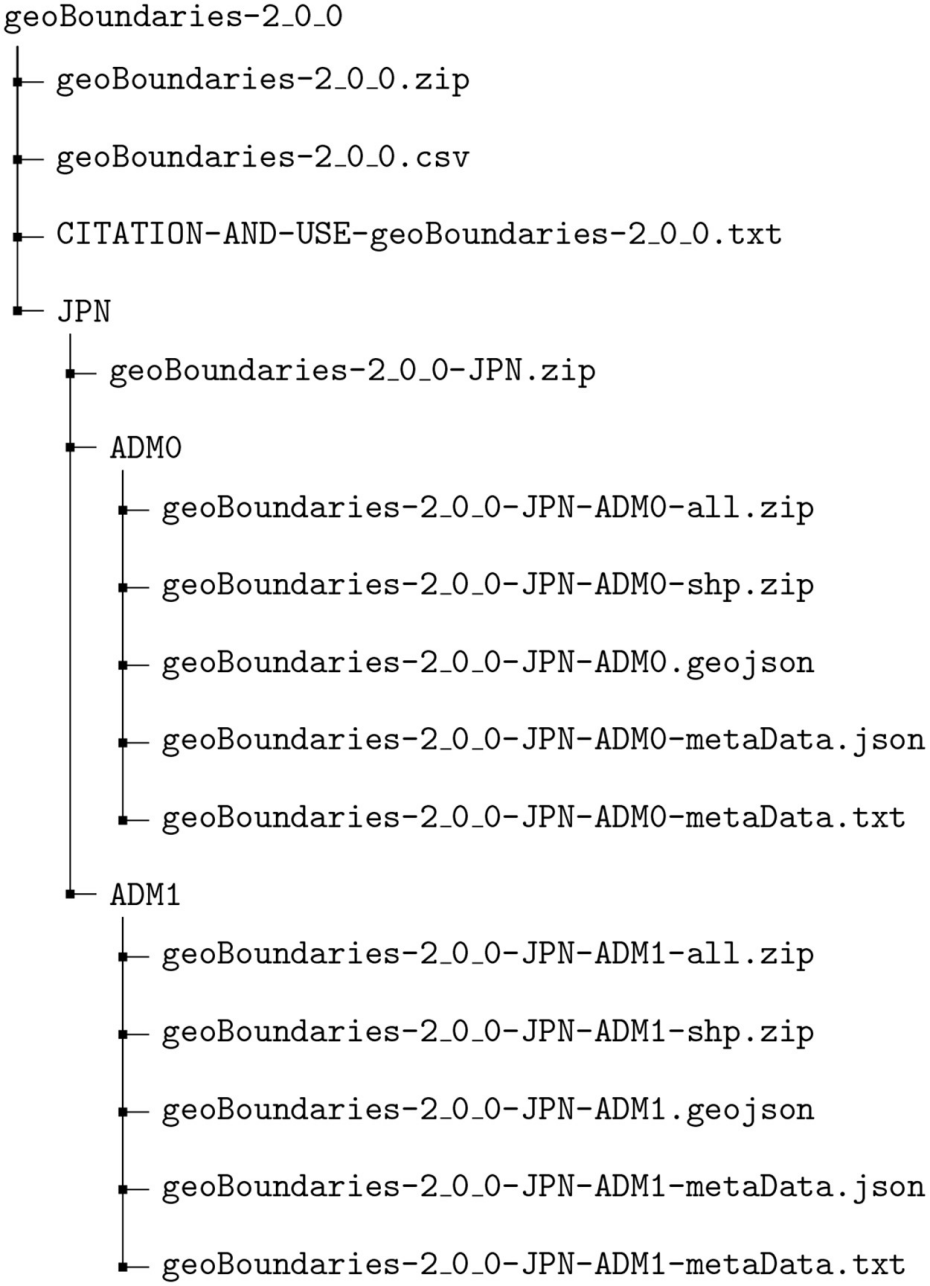
Example file structure of the geoBoundaries data product. This structure can be used to construct a download URL for any file in the database—for example, https://geoboundaries.org/data/geoBoundaries-2_0_0/JPN/ADM0/geoBoundaries-2_0_0-JPN-ADM0-shp.zip can be used to download the shapefile for the specified country and ADM level.

After these schema standardization steps for each boundary group and shape, we generate four files which are deposited into the appropriate path *P*_*i*_ for each boundary. These include: (1) a zipped version of a shapefile and accompanying files necessary for use; (2) a stand-alone GeoJSON, (3) a human-readable text file (*.txt) containing the relevant metadata for each boundary, and (4) a machine-readable JSON containing the same metadata information. Finally, the contents of every folder are recursively zipped into single files for user convenience. This file hierarchy is mirrored onto an online repository for public consumption. The resultant file structure end-users will observe is shown in Fig 2.

In addition to provisioning files following this URL-based approach, we also provide access via a programmatic API. The API allows an end-user to automatically request the path to the latest version of a geoBoundary by calling (as an example):

http://www.geoboundaries.org/gbRequest.html?ISO=AFG&ADM=ADM0

This API will return a JSON that contains all metadata for the most recent version of the requested geoBoundary, including the ‘downloadURL’ field and the most recent date of update. Further, the special keyword ‘ALL’ can be specified for either the ISO or ADM to retrieve all boundaries from a country or hierarchy. Users seeking programmatic access into this database can leverage this to automatically check for updates and retrieve relevant boundary geometries for their own use cases.

### Validation

All boundary data is collected from government published or reliable internet sources; in cases where an authoritative source is not available we have identified at least 2 sources indicating boundary information is accurate. We further apply a wide range of both manual and automated quality assurance checks and corrections, as described above. Researchers interested in contributing to this project are encouraged to contact the corresponding author; we will accept data from published sources (e.g., scientific papers) so long as it adheres to the schema and quality standards outlined in this document. In cases where boundaries may disagree, we will publicly engage in conversations around which boundaries to include in our releases, and ensure that we provide links to alternative boundaries even if they are not selected for inclusion in the main database so as to facilitate the potential comparison of contrasting perspectives of geographic boundaries. As a public and evolving source of data, geoBoundaries consistently incorporates changes or improved source information based on user contributed suggestions.

## Results & discussion

Following the procedures outlined above, 351,819 individual shapes delineating legal boundaries were collected, processed, and prepared for distribution. Table 3 shows the count of each license type currently in the geoBoundaries database; the vast majority (402) are released pursuant to the Open Data Commons Open Database License 1.0.

**Table 3:** A summary of license types currently included in the geoBoundaries dataset. Explicit detail on the license for every boundary is provided in the metadata.

License Name (Source)	Count of Boundary Sets
Open Data Commons Open Database License 1.0	402
Creative Commons Attribution (Various Versions)	61
Public Domain	12
Open Government Licence (v3.0 and v1.0)	7
MIMU Data License (MIMU)	4
Other License Types	131

Despite the advance this piece represents—the first open and redistributable set of administrative geographic boundaries curated explicitly for scientific precision and replication—we note that the range of open boundary licenses currently included in our database could still preclude some uses. For example, while the Open Government License is very similar in permissiveness to the Creative Commons and Open Data Commons licenses, we acknowledge that our users may not have the time or capability to determine if every license meets their particular use case. Our core goal as we continue to improve this data source is to harmonize all licenses; however, we note that such an endeavor may yet take years. Further improvements we seek to provision include an expansion to higher levels of granularity in administrative hierarchies, additional precision in boundary files, and a gradual expansion of our boundary data into a time series format.

As large-scope analyses become more common, data sources such as the one presented here will become increasingly critical to support open discussion around scientific findings. The geoBoundaries database provides a meaningful pathway forward for researchers seeking to promote the replication of analyses that leverage administrative boundary data, from country to global scales.
